# Behaviors of Glioblastoma Cells in *in Vitro* Microenvironments

**DOI:** 10.1038/s41598-018-36347-7

**Published:** 2019-01-14

**Authors:** Wenwen Diao, Xuezhi Tong, Cheng Yang, Fengrong Zhang, Chun Bao, Hao Chen, Liyu Liu, Ming Li, Fangfu Ye, Qihui Fan, Jiangfei Wang, Zhong-Can Ou-Yang

**Affiliations:** 1 Key Laboratory of Theoretical Physics, Institute of Theoretical Physics, Chinese Academy of Sciences, 55 East Zhongguancun Road, Beijing, 100190 China; 2 School of Physical Sciences, University of Chinese Academy of Sciences, No. 19A Yuquan Road, Beijing, 100049 China; 3 Department of Neurosurgery, Beijing Tiantan Hospital, Capital Medical University, Beijing, 100050 China; 4 Beijing National Laboratory for Condensed Matter Physics and CAS Key Laboratory of Soft Matter Physics, Institute of Physics, Chinese Academy of Sciences, Beijing, 100190 China; 5 Wenzhou Institute of Biomaterials and Engineering, Chinese Academy of Sciences, Wenzhou, 325001 China; 6 School of Optometry and Ophthalmology and Eye Hospital, Wenzhou Medical University, Wenzhou, 325027 China; 7 College of Physics, Chongqing University, Chongqing, 401331 China

## Abstract

Glioblastoma (GBM) is the most malignant and highly aggressive brain tumor. In this study, four types of typical GBM cell lines (LN229, SNB19, U87, U251) were cultured in a microfabricated 3-D model to study their *in vitro* behaviors. The 3-D *in vitro* model provides hollow micro-chamber arrays containing a natural collagen interface and thus allows the GBM cells to grow in the 3-D chambers. The GBM cells in this model showed specific properties on the aspects of cell morphology, proliferation, migration, and invasion, some of which were rarely observed before. Furthermore, how the cells invaded into the surrounding ECM and the corresponding specific invasion patterns were observed in details, implying that the four types of cells have different features during their development in cancer. This complex *in vitro* model, if applied to patient derived cells, possesses the potential of becoming a clinically relevant predictive model.

## Introduction

Malignant gliomas are the most common primary brain tumors^1^, among which glioblastoma (GBM) is the most malignant and highly aggressive, belonging to grade IV gliomas according to the World Health Organization (WHO) classification system^2,3^. The median life expectancy for GBM patients is only 12–15 months even with a treatment combining resection, radiation therapy, and chemotherapy^4,5^. GBMs can recur within 1–2 cm of the primary tumor border^6^. One major cause of treatment failure and tumor recurrence is diffuse invasion of GBM cells into the surrounding brain tissue^6,7^. Therefore, it is critical to understand the invasion mechanism of GBM cells, in order to devise efficient therapeutic strategies.

Given that *in vivo* animal models are complex, expensive, time consuming, various *in vitro* models have been constructed to further study the complex interactions between GBM cells and extracellular matrix (ECM)^4,6,8–14^. Cells cultured in traditional two-dimentional (2-D) models (on Petri dish or on hydrogel substrates) can produce fast response to environment modulation, but the microenvironment for cells in 2-D models is quite different from *in vivo* conditions^15–17^, and there is no 2D model that can provide *in-vivo*-like three-dimensional (3D) confinement by ECM. Three-dimensional models have been developed to mimic the brain tissue for GBMs research. On low adhesion surface or in suspension, freshly isolated GBM cells from patients can be cultured as clonally dividing neurospheres in serum-free, xeno-free medium^18,19^. The neurosphere cultures form a semi-3-D *in vitro* model, while maintaining the stemness of GBM cells^4,20^. However, neurospheres usually need a longer preparation process. To better mimic the *in vivo* microenviroment, hydrogels, in particular, natural hydrogels extracted from animals (such as collagen)^21^, have been introduced as a substitution of native ECM for *in vitro* models due to their high water content and proper mechanical properties. GBM cells or fragments of tumour are directly embedded and grow in hydrogel to form *in vitro* 3-D models^21–25^. These 3-D models can simulate the diffusion of nutrients and oxygen through tissue, and can be used for studies of cell invasion through native ECM. Cell tests in 3-D models often show dramatically different results from those in 2-D models^26,27^.

**Figure 1:**
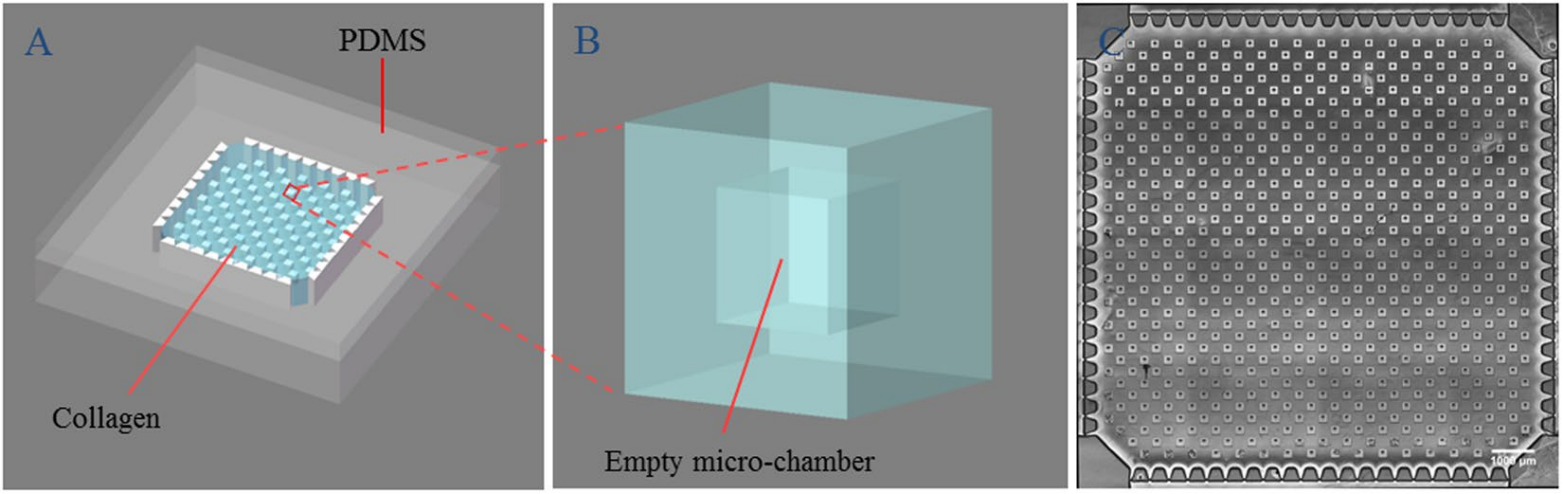
(**A**) 3D *in vitro* model with micro-chambers in collagen; (**B**) enlarged image showing one micro-chamber; (**C**) top view of the chip, captured in the bright field and processed with ImageJ, where the scale bar is 1000 μm. The size of the microchambers is 200 × 200 μm^2^, with the distance between nearest neighbor pairs being 400 μm.

In this article, in order to better understand the metastasis of GBMs, in particular, the interaction between GBMs and ECM, four types of GBM cells lines (LN229, SNB19, U251, U87) with origin from neuroepithelial cells were cultured in a micro-fabricated 3-D *in vitro* model, and their behaviors were thoroughly studied. The micro-structured chips in the model were constructed to possess an array of 3-D hollow micro-chambers embedded in collagen I gel, as shown in Fig. 1, so as to enable *simultaneous* investigation of GBM cells’ proliferation, migration, and invasion in a suitable microenvironment^28–30^. The micro-chambers in the collagen can provide a fully natural-like interface for glioma cell to attach, proliferate, and even invade into surrounding ECM as *in vivo* conditions, without the interference of any solid substrate, which may change the cell behavior. The analysis based on our *in vitro* model can provide many details for gliomas metastasis study. For example, glioma cells usually invade as individual cells, which are responsible for tumour recurrences but undetectable by most sophisticated diagnostic imaging techniques^31^. In our *in vitro* model, this single cell metastasis process can, however, be observed and well analyzed. Furthermore, this micro-constructed *in vitro* 3-D model has several advantages in mimicking and observing *in vivo* behaviours of GBM cells. Firstly, it can be used for the study of tumour cells and ECM interaction, and has a potential of mimicking complex tumour microenvironment. Secondly, the transparency of this 3-D model allows the study of the entire process of cell migration and invasion. Thirdly, the presence of hundreds of micro-chambers in each chip enables high-throughput cell tests. With the benefit of this 3-D microfabricated *in vitro* model, we uncover some phenomena of the four GBM cell lines in the aspects of morphology, proliferation and invasion, which may be related to GBMs’ clinical behaviors. This complex *in vitro* model, if applied to patient derived cells, could also potentially become clinically useful predictive models^31^.

## Results

### Morphology of GBM cells in the 3-D model

**Figure 2:**
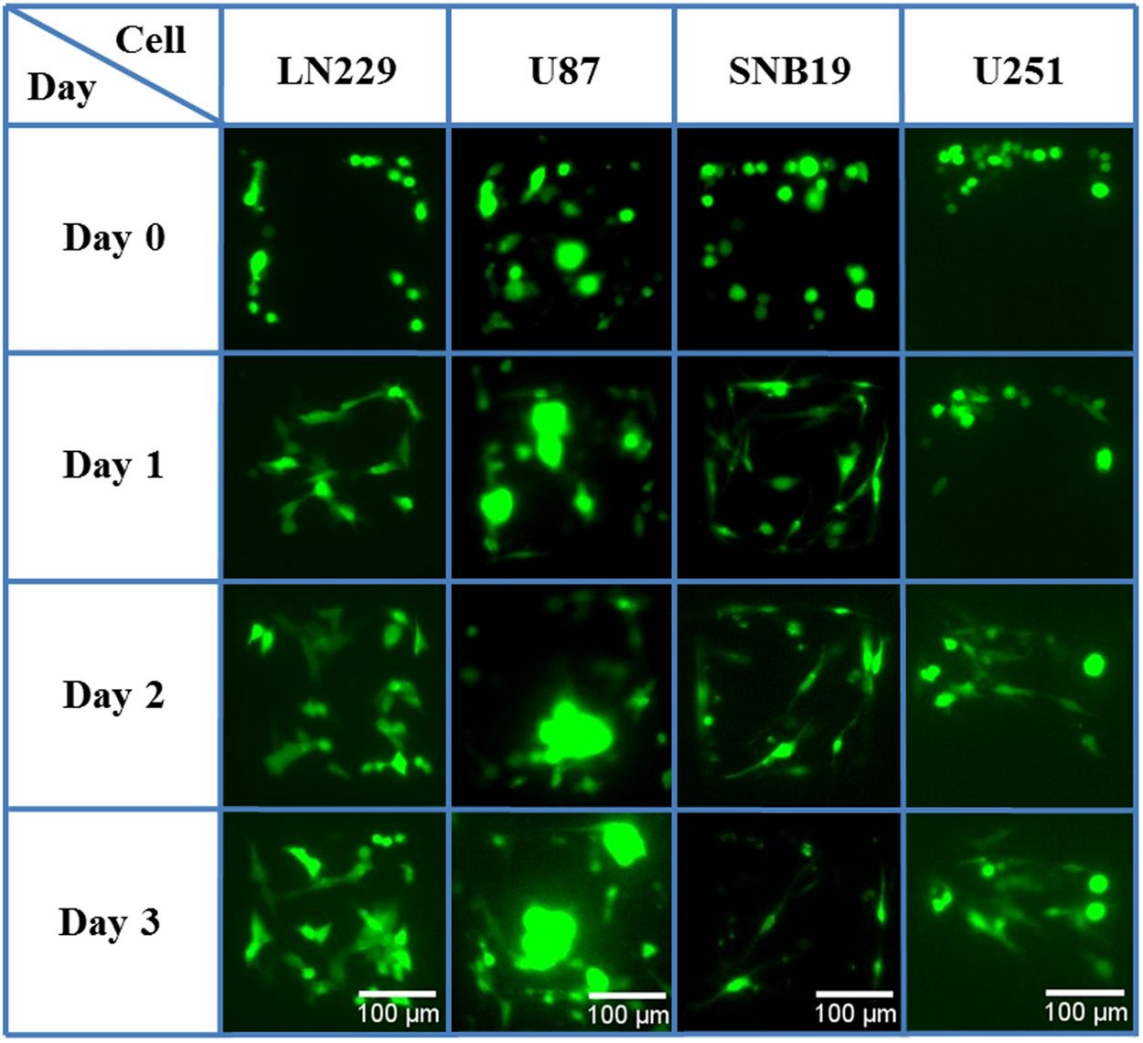
Representative fluorescent images showing the morphology of the four types of GBM cells in micro-chambers within collagen during the four culture days.

Each type of GBM cells were seeded in the micro-chambers within collagen on Day 0. Cell morphology was observed for the following three days (Day 1 to Day 3). Different types of GBM cells showed dramatically different behaviors in the micro-chambers, as shown in Fig. 2. The shapes of LN229 (Supplementary Video 1) were triangular or diamond, and those of the SNB19 cells were slender, similar to their respective shapes in the case of being cultured on a 2-D surface of Petri dish (see Fig. S3 in Supplementary Information). The U251 cells had irregular shapes and long protrusions, some of which invaded out of the micro-chambers on Day 3 (Supplementary Video 4). Fig. S3 also shows the cells in high magnification and low cell density. From the bright field images, fluorescent images, and dynamic videos (Supplementary Videos 7–10), we can tell the cell shape and migration in more details.

Regarding the U87 cells, they moved very fast and then aggregated as clusters in one day (Supplementary Fig. S8). The size of the U87 cell clusters kept increasing during the following days. Starting from Day 2 and Day 3, some cells and even cell clusters invaded out of the chambers (Supplementary Fig. S8). The 3-D confocal microscope images clearly show that the U87 clusters reached the top of the micro-chambers and formed a neurosphere-like structure (Supplementary Video 6). Note that the cell culture medium for this 3-D model contains serum; in contrast, the culture medium of growing neurospheres in solution (without any gel) should be depleted of serum.

### Proliferation rate of GBM cells in the 3-D model

To quantify the cell proliferation rate, fluorescent images of cells (including both the cells in individual chambers and those invading into the surrounding gel) were acquired from Day 0 to Day 3 with an inverted fluorescent microscope (Nikon Ti-E). The integrated intensity of fluorescent cells in each micro-chamber was analyzed using ImageJ software (http://imagej.nih.gov/ij/) and used as an indicator of cell numbers. All the images for measuring the integrated intensity of cells were captured with the same setting parameters, including the light intensity of exciting light, exposure time, etc. Then the images were processed with the software ImageJ, also with same setting values. For example, contrast and brightness of all the images were processed with the same settings. After that, the images were transformed to binary images with the same threshold value. Then the integrated intensity of cells was measured by the software directly. Because the capture and image processing settings were all kept the same, we believe that the extracted integrated intensity of cells is reasonable.

To validate the precision of this technique, a control experiment has been conducted. Cells with different densities were seeded in the micro-chambers, and the integrated intensity was measured on Day 0. As shown in Fig. S10, the normalized integrated intensity of the cells is linearly proportional to the cell numbers, implying the integrated intensity can be used as an indicator of cell numbers.

Because U87 tends to form neurosphere-like structures, nuclei staining helps us to verify the proliferation of U87. As shown in Fig. S4, U87 nuclei were stained with Hoechst (Life technologies) before seeding into micro-chambers for culture. The proliferation of U87 was quantified via both nuclei counting and the integrated intensity of fluorescent protein in cells. The analytical result via nuclei counting is consistent with the cell proliferation data via measuring the integrated intensity, which also verifies the accuracy of the analysis techniques.

**Figure 3:**
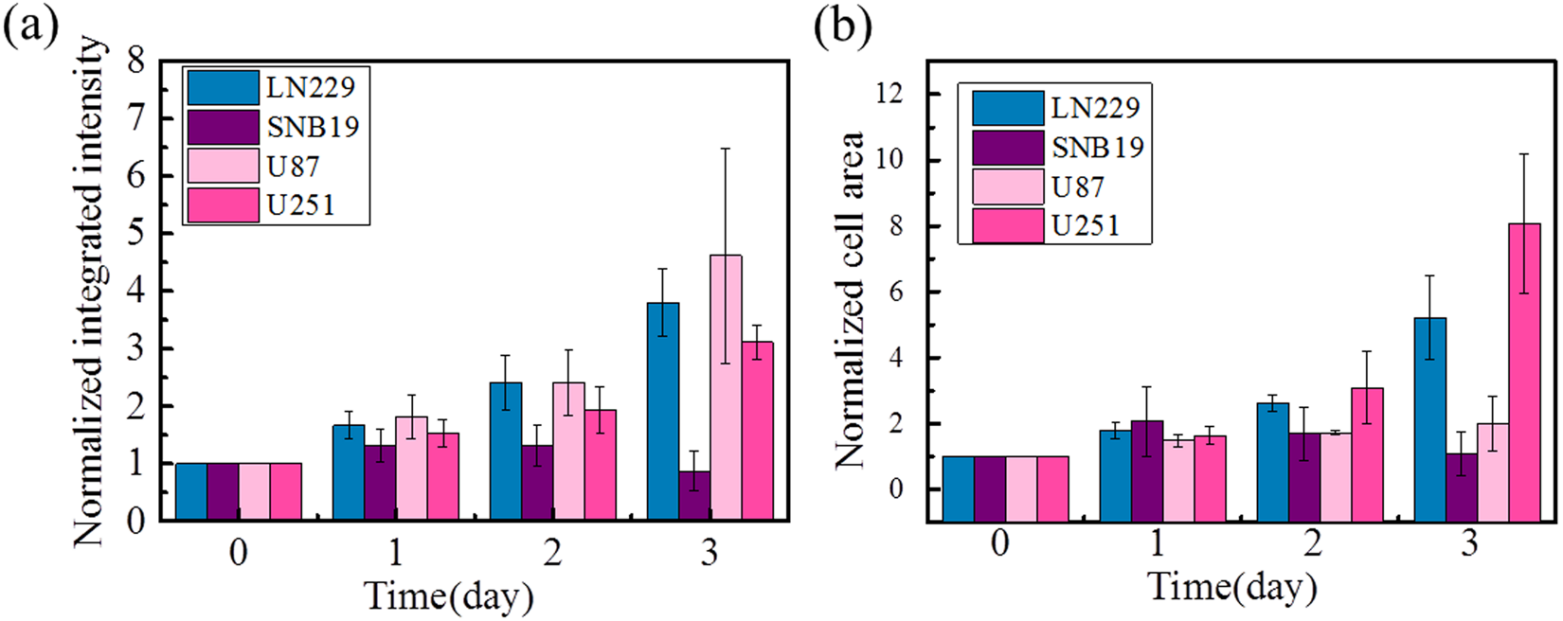
Cell proliferation rate of LN229 (blue), SNB19 (purple), U87 (light pink), and U251 (dark pink) indicated by (**a**) normalized integrated intensity and (**b**) normalized cell area. Data are presented as mean ± SD of three independent experiments.

The cells in at least 8 micro-chambers per sample were monitored, and 3 parallel samples for each cell line were analyzed. For each sample, the analyzed integrated intensities of the four culture days were normalized to the results of Day 0 to get the data of relative cell numbers, indicating how fast the cells proliferated. Figure 3a shows that the (normalized) integrated intensities of LN229, U87 and U251 keep increasing during the four culture days. The proliferation rates of LN229, U87 and U251 are higher than that of SNB19; and among the former three, the proliferation rate of U251 seems smaller than those of the other two. The numbers presented here include both the cells in individual chambers and those invading into the gel. The SNB19 cells proliferated rapidly for the first two days, but decreased dramatically after Day 2, maybe because of their failure to adapt to the environment after invading into collagen (Supplementary Fig. S7).

Due to the variance of the fluorescent emissions from each cell and the variance of the cell shape of different cell lines, one more parameter was measured together with the integrated intensity to analyze the cell proliferation rate. This additional parameter is the total area of fluorescent cells in each chamber. Figure 3b shows the characterization of cell areas during the four culture days. Similar to the integrated intensity characterization result, the normalized cell areas of LN229, U87 and U251 cells also increased. However, the proliferation of U251 cells seems faster than those of LN229 and U87, if indicated by cell areas, which is contrary to the result indicated by the integrated intensities. This is probably because the U251 cells have higher deformability and are more spread out. Interestingly, although the U87 cells proliferated rapidly as indicated by the integrated intensity, the cell area of U87 cells increased very slowly, indicating the formation of U87 clusters which expanded to the largest extent in z direction rather than spreading on the surface.

We also conducted the control tests seeding cells with densities of about 125 cells/mm^2^ and 625 cells/mm^2^ to investigate if the initial cell density would impact the cell activity. From the statistical results, as shown in Fig. S14(a–d), the initial cell density is not very critical in impacting the overall cell behavior. The cell proliferation rates for high and low densities of seeding cells are similar to each other during the first three days. There is only one exception: when the initial cell density is very low, the U87 cells cannot get together and form clusters. This may be because clustering facilitated the proliferation of U87 cells while the chambers decreased the proliferation rate of the isolated U87 cells.

### Migration of GBM cells in micro-chambers confined within collagen

Cell migration is a very important process associated with cancer progression. Cell migration and persistence in the hollow micro-chambers within natural hydrogel collagen are investigated to reveal migration features in brain tumor. For each cell line, at least 20 cells were tracked, and three parameters were analyzed: speeds, directionality ratio, and mean square displacement (MSD). Here, the speed is measured by software Itrack4U or CellTracker; it reflects the cell motility in short time intervals. The directionality ratio is obtained by dividing the displacement by the trajectory length; it is commonly used by biologists to characterize the persistence of cell migration. The MSD contains information about both the speed and the persistence; and the value of the MSD itself also represents the area a cell can explore on average.

In the experiments, six hours after the cell seeding, the chip was placed into an on-stage incubator, which maintained a humid environment with 37 °C and 5% CO_2_ during the entire recording process under microscope. Time lapse images for each sample were captured every 15 minutes for 12.25 hours by using an inverted fluorescent microscope. The movement of individual cells in the micro-chambers can be tracked with software Itrack4U or CellTracker by analyzing the captured time-lapse images^32^. The cells that divided or migrated out of the field of view during the tracking period were excluded from analysis. The corresponding results are given below.

**Figure 4:**
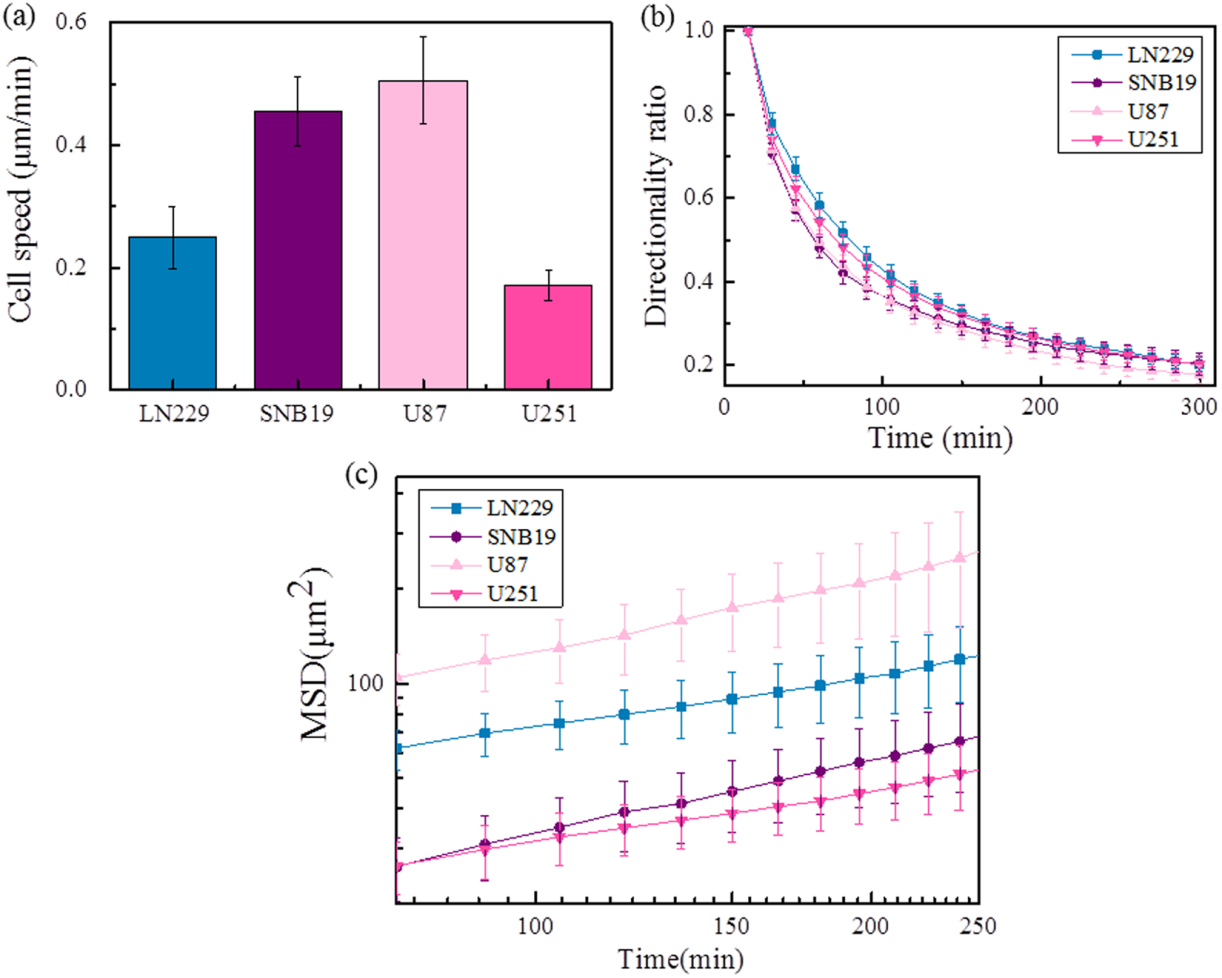
Cell migration speed, directionality ratio, and MSD of four types of GBM cells: (**a**) the average cell migration speed (N = 200 for each test); (**b**) directionality ratio (N = 20 for each test); (**c**) mean square displacement (MSD), plotted as function of time interval (N = 20 for each test). Data are presented as mean ± SD of three independent experiments.

The U87 cells have the highest average migration speed (0.50 ± 0.07 μm/min) among the four types of GBM cells, as shown in Fig. 4a. The migration speeds of SNB19 cells and LN229 cells are lower, with an average speed of 0.45 ± 0.05 μm/min and 0.25 ± 0.05 μm/min, respectively. The motility of U251 cells is very low, with an average speed of only 0.17 ± 0.02 μm/min. However, for t < 100 mins, the LN229 cells have higher directional ratio than the other three types of cells, as shown in Fig. 4b. Note that the speeds given here are from measurements carried out for cells seeded on the surface of collagen gel, rather than the cells in the chambers. By doing so, we aim to exclude the potential influence from the chamber walls on the speed measurements.

The value of α in$\,\mathrm{MSD}({\rm{t}})\,\propto \,{({\rm{t}})}^{\alpha }$ allows us to distinguish between random diffusion (α = 1), subdiffusion (α < 1), superdiffusion (α > 1) and a stationary process (α = 0)^33,34^. Linear fitting of our MSD data in the 75–250 minutes gives the α value of LN229, SNB19, U87, U251 cells, which were respectively 0.5385 ± 0.0088, 0.7776 ± 0.0075, 0.7457 ± 0.0126, 0.5549 ± 0.0112. The α’s of all four GBM cells are lower than 1, indicating that the cell motion is in a subdiffusion state, probably because of the confinement of the chambers. The α values of U87 and SNB19 are higher, indicating they probably have higher persistance and tend to invade into the collagen, which is consistent with the invasion ability tests via the protrusion length analysis in the following part.

We have done the cell migration tests on the petri dish as in the traditional 2-D system, using all these four types of cells. The cell migration speed and other statistical analysis have been added in the supplementary information, as shown in Fig. S6. In the 2-D system, LN229 cells have the highest average migration speed (0.31 ± 0.07 μm/min) among the four types of GBM cells, as shown in Fig. S6(a). The migration speeds of SNB19 cells and U87 cells are lower, with an average speed of 0.30 ± 0.09 μm/min and 0.24 ± 0.07 μm/min, respectively. The motility of U251 cells is very low, with an average speed of only 0.10 ± 0.05 μm/min. Linear fitting of our MSD data in 75–250 minutes gives the α value of LN229, SNB19, U87, U251 cells, which were respectively 1.158 ± 0.0027, 0.9376 ± 0.0065, 1.1329 ± 0.0070, 1.0139 ± 0.0093 [Supplementary Fig. 6(c)]. The α values are all around 1, indicating the cells migrate randomly.

A control test studying the influence of the cell density on the cell migration speed has also been conducted. Fig. S14(e) compares the results for the density of ~125 cells/mm^2^ with those for the density of ~625 cells/mm^2^, indicating the initial cell density is not very critical in impacting the cell migration speeds.

### Invasion of GBM cells into the surrounding collagen

**Figure 5:**
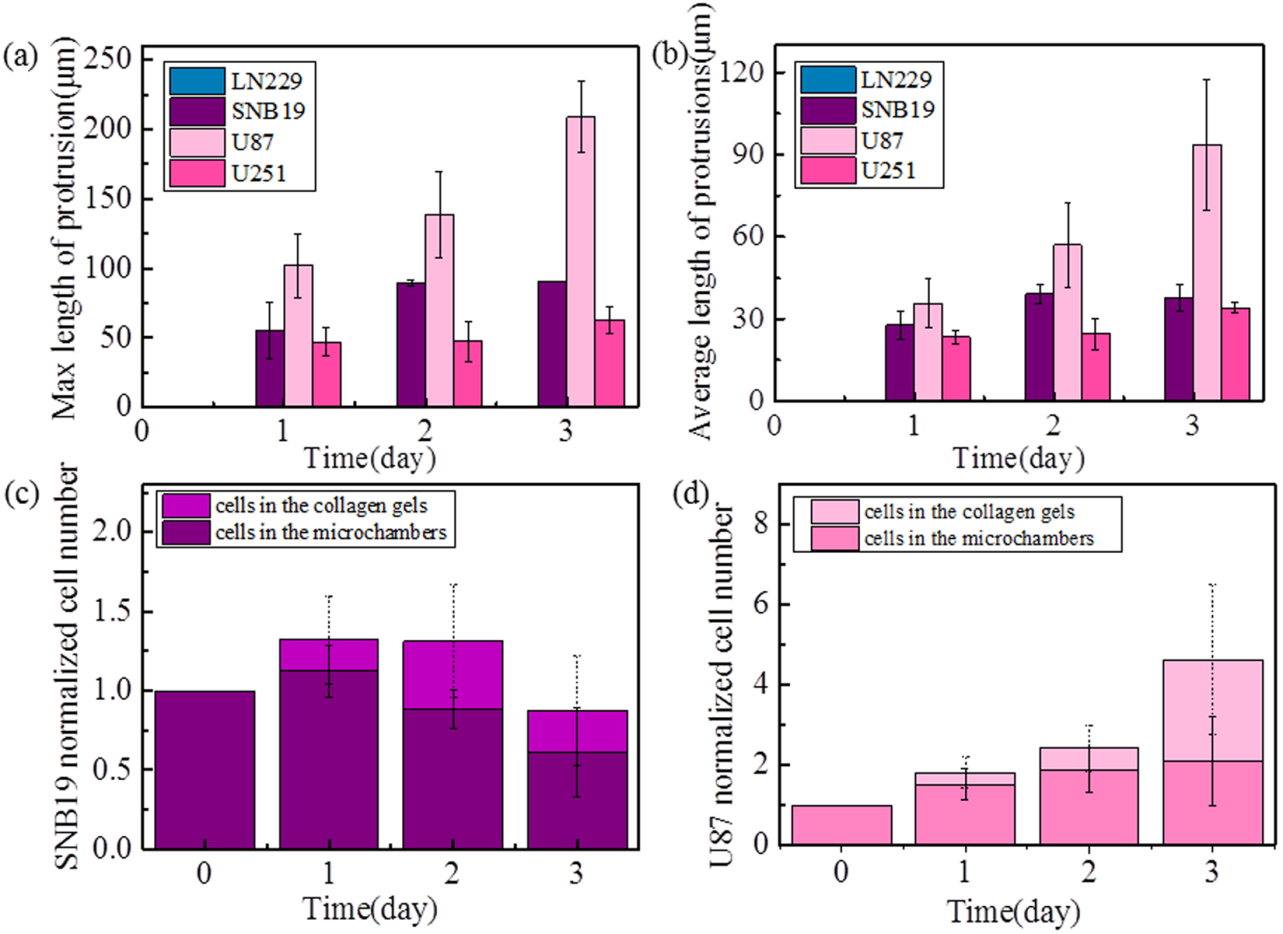
(**a**) Average and (**b**) maximum protrusion length of four types of GBM cells; (**c**) normalized cell number (conjectured from normalized integrated fluorescent intensity) of SNB19 cells inside individual micro-chambers and those invading into the surrounding collagen gel for four consecutive days; (**d**) normalized cell number (integrated intensity) of U87 cells inside individual micro-chambers and those in the surrounding gel. Data are presented as mean ± SD of three independent experiments.

To explore the process of GBM invasion in detail, cells were cultured in the hollow micro-chambers within collagen, and were observed to study their interaction with collagen. The ability of cell invasion is indicated by three parameters: the average length of filopodia protrusions, the maximum length of protrusions, and the number of cells completely invading into the collagen gel. To obtain the average length of protrusions of GBM cells, only the protrusions whose corresponding cell bodies were still in the microchambers were measured. At least 8 micro-chambers in each of the three parallel samples were monitored for four days. The total length of all protrusions per sample was measured via ImageJ and then divided by the corresponding total cell numbers to give the average protrusion length. The second parameter to characterize the cell invasion ability is the length of the longest protrusion in each sample. The third parameter is the number of cells which entirely invaded out of the micro-chamber, defined as those with the entire cell body invading into the surrounding collagen. The size of the micro-chambers was 200 × 200 μm^2^. We defined a larger region surrounding each micro-chamber, with an area of about 260 × 420 μm^2^, to include all the GBM cells originally from the micro-chamber. The cells entirely invading out are those invaded out of the micro-chamber but still in the surrounding larger region. Then, the integrated intensity from fluorescent cells in these two areas was measured and normalized to the result of the first day to indicate the relative cell numbers of SNB19 and U87 in Fig. 5(c,d).

The data of the average protrusion length and maximum protrusion length given in Fig. 5(a,b) indicate that U87 cells have longest protrusions into the collagen. The average protrusion length of the U87 cells even reached 93.6 μm on Day 3. The SNB19 cells and U251 cells also formed protrusions in the collagen at the beginning of Day 1. However, these protrusions grew very slowly in the following days. Especially, the SNB19 cells in the micro-chambers died gradually after Day 3. The LN229 cells did not start to extend small protrusions into collagen until Day 5 when the micro-chamber was completely filled with cells (Supplementary Fig. S9).

**Figure 6:**
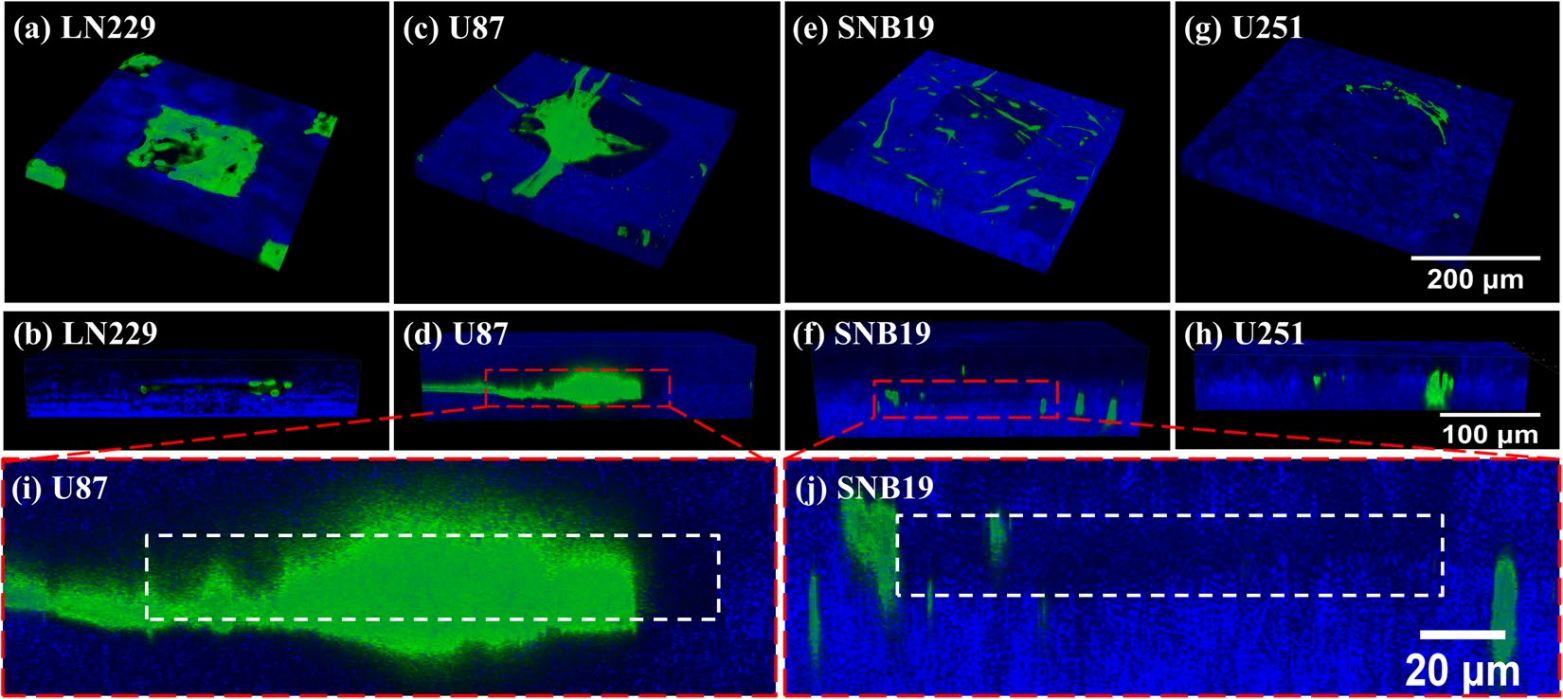
Confocal images showing representative cell filopodia protrusions (green fluorescent) in a single micro-chamber, where the top and bottom rows are, respectively, top-view and side-view images. (**i**,**j**) Locally amplified side-view images of (**d**) and (**f**). The collagen fibers (blue) were observed in reflection mode.

The filopodia protrusions from cells were observed through a confocal laser scanning microscope to reveal their invasion into the surrounding ECM. The invasion patterns of different cell types vary dramatically. Figure 6(a) shows the LN229 cells exhibited no obvious invasion into the collagen on Day 3. The U251 cells remained in the chambers although they protruded into the gel. The SNB19 cells showed a behavior of single cell invasion [Fig. 6(e,f)], with the ability of invading deeply to the bottom of the collagen micro-chamber (Supplementary Fig. S16 and Video 5). On the contrary, the U87 cells invaded as clusters, i.e., in a collective manner (Supplementary Fig. S17 and Video 6), with a bundle of filopodia protrusions from multiple cells in the cluster forming thick protrusions extending from the cluster and invading into the surrounding collagen.

## Discussion

In this study, four types of typical malignant GBM neuroepithelial cells (LN229, SNB19, U87, U251) were cultured in hollow micro-chambers within collagen, and the corresponding properties on the aspects of cell morphology, proliferation, migration, and invasion were compared. This microfabricated 3-D *in vitro* model provides a well suited microenvironment for cell study due to the use of natural hydrogel (i.e., collagen gel) and the presence of the 3-D hollow room which provided a natural surface for growing cell. Furthermore, in this model, GBM cells’ proliferation, migration, and invasion can be investigated simultaneously, and the dynamical process and migration pattern associated with GBM cells’ invasion into the surrounding ECM can be observed in detail.

Our results present some properties not observed in previous 2-D or simple 3-D environments. Following collective behaviors, U87 cells showed rapid migration, and highest invasion ability (in terms of the length of protrusions into the surrounding collagen and the number of cells invading into the collagen). The SNB19 cells started to invade at an early stage, but showing only individual invasion. The LN229 cells proliferated rapidly, but did not start to invade until the micro-chamber was fully occupied by the cells, i.e., their invasion was probably driven by the population pressure. With a large spreading area, the U251 cells migrated slowly and invaded the collagen mainly through filopodia protrusions. Our experimental results thus indicate that the GBM cells we observed can be classified into three distinct types: expansive-growth cells (including LN229 and U251), whose proliferation rate is large and invasion ability is weak, individual invasion cells (SNB19 cells), and cluster invasion cells (U87 cells)^35,36^.

We emphasize that all the parameters analyzed in this model are related to the invasion ability of cancer cells. The cell morphology and migration mode represent the invasion mode, i.e., the single cell invasion (SNB19) or collective cell invasion (U87). The proliferation rate of cells is critical to cancer malignancy. The migration speed and persistence are partially related to the capability of cell invasion. The protrusions of cancer cells into collagen directly show the invasion behaviors.

These specific properties of each type of GBM cells may reflect their metastasis behavior *in vivo*. For example, it has been reported that a xenograft tumor volume of LN229 cells grew at a relatively fast rate and led to vascularization of the tissue^37,38^; U251 xenografts were observed to mainly increase in tumor size^39,40^; U87 implantation was reported to show large well-defined masses in the brain parenchyma, with rare single cell invasion^41–43^.

In near future, we will culture primary GBM cells or organoids from patients in this 3D *in vitro* model, aiming to provide a reference for clinical therapy design. The organoids can be obtained by hanging drop technique. Once the organoids have formed, they’ll be picked up and seeded into the micro-chambers via the assistance of micro-pipettes. After seeding the organoids, the other procedures are the same as those processing single cells. Given that the collagen and PDMS pieces of this model are almost transparent, primary cells and organoids can be clearly observed directly via bright-field microscopy without any fluorescent tag. If necessary, multiple fluorescent bioprobes can be adopted to mark the cells rapidly.

## Materials and Methods

### Cell culture and extracellular matrix preparation

Human GBM cell lines U87, U251, LN229, and SNB19 were obtained from the Institute of Biochemistry and Cell Biology, Chinese Academy of Science (Shanghai, China). Green fluorescent proteins (GFP) were transfected into GBM cells by lentivirus infection method. Cells were maintained in Dulbecco’s modified Eagle’s medium-F12 (DMEM/F12, Gibco) supplemented with 10% fetal bovine serum (Gibco) and 50U/ml penicillin, and 250 μg/ml streptomycin (Corning). All cells were cultured in an incubator at 37 °C with 5% CO_2_.

Neutralized collagen solutions were prepared by mixing buffer solutions of sterile deionized water, 10X PBS (Corning) and 1 mol/L NaOH (Fluka) with the high concentration collagen (Corning) on ice, according to the manufacturer’s instructions^44^. The final collagen concentration was 6 mg/mL for all the tests.

### Fabrication of the micro-structured 3-D *in vitro* model

PDMS components for the 3-D *in vitro* model were fabricated using the traditional lithography technique^30^. The fabricated PDMS substrate was pre-coated with 1 mg/ml fibronectin solution and air-dried completely before being used to enhance the adhesion with collagen. The PDMS stamp was pre-coated with 3% bovine serum albumin (BSA, Beyotime), allowing the stamp to get off of the collagen gel. Each PDMS stamp was aligned with a PDMS substrate. The prepared neutralized collagen solution was injected into the collagen area in the PDMS substrate through a pre-drilled inlet. Collagen top layer was prepared by pipetting 30 μl of neutralized collagen solution in the center of a sterile coverslip. The PDMS substrate with injected collagen and the collagen top layer were incubated in a 37 °C incubator for at least 3 hours to gel. After gelling, PDMS stamp was peeled off carefully, leaving the collagen substrate with patterned micro-wells. The gelled collagen top layer was covered on top of the fabricated collagen substrate to form sealed hollow micro-chambers^45^. Confocal images showed perfect micro-chambers on Day 0 in Supplementary Fig. 11.The schematic diagram of the method is shown in detail in Fig. S1 of Supplementary Information.

### Cell seeding

A single-cell suspension with density of 1 × 10^7^ cells/ml was prepared right before the experiment. 50 μl of the pre-chilled cell suspension was added onto the surface of the collagen substrate. After 1-2 minutes when the micro-wells were filled with cells, the cells outside the micro-wells were rinsed off with cold 1× PBS flow (flow rate 100 μl/min) (Supplementary Fig. S2). The collagen substrate was then incubated at 37 °C for 5 min to allow the cells to adhere to the collagen. The cell-seeded collagen substrate was then covered with the collagen top layer. The fabricated chip was put in a sterile Petri dish, and filled with cell culture medium.

### Confocal microscopy

Cells and collagen matrices were imaged with a confocal laser scanning microscope (CLSM, Leica SP8) with HCX IRAPOL 25×/0.95 WATER immersion objective. The pinhole is set to 1.0 airy unit. Under the excitation light of 488 nm, a PMT detector collects the signals from the wavelength of 480 nm to 498 nm for cell imaging. Meanwhile, the reflection mode of the CLSM was used to image the collagen fibers, as in previous studies^46^. All images within one experiment were taken with the same parameters to keep consistence. We use confocal microscopy to capture successive two-dimensional slices at different z-axis positions in a sample. The confocal microscopy enables the reconstruction of three-dimensional structures of an object. We can also transect the reconstruction in different axes to get the top-view and side-view images.

### Computation of directionality ratio and mean square displacement

The directionality ratio is defined as follows,

1 $$\mathrm{Directionality\; ratio}=\langle \frac{{{\rm{d}}}_{{\rm{n}}{\rm{\Delta }}{\rm{t}}}}{{{\rm{D}}}_{{\rm{n}}{\rm{\Delta }}{\rm{t}}}}\rangle $$

where Δt is the minimal time interval (Δt = 15 minutes) between two consecutive images of the tracked cell, d_nΔt_ denotes the displacement between the initial point, the current point at time nΔt, D_nΔt_ is the actual length of the cell trajectory corresponding to d_nΔt_, and the bracket indicates that the directionality ratio is averaged over at least 20 cells for each kind of GBMs.

Mean square displacement (MSD), whose relation with time shows whether cells’ migration belongs to diffusion, subdiffusion, or superdiffusion^33^, is computed by the following equation^47^:

2 $${\rm{MSD}}(n{\rm{\Delta }}t)=\frac{1}{N-n+1}{\sum }_{i=0}^{N-n}[{({x}_{(i+n){\rm{\Delta }}t}-{x}_{i{\rm{\Delta }}t})}^{2}+{({y}_{(i+n){\rm{\Delta }}t}-{y}_{i{\rm{\Delta }}t})}^{2}]$$

where N is the total number of steps in a trajectory. Note that overlapping time intervals has been employed in the above equation. The MSDs given in Eq. (2) are for a single trajectory, and are then averaged over at least 20 cells of each kind of GBMs to obtain a population-average value.

### Data analysis and statistics

For each experiment, at least three samples were prepared, and at least eight micro-chambers in each sample were selected. The error bars indicate the standard deviations (SD) of the data resulting from measuring various micro-chambers. All values are expressed as the mean ± SD.

## Electronic supplementary material

Supplementary Information

Supplementary Video 1

Supplementary Video 2

Supplementary Video 3

Supplementary Video 4

Supplementary Video 5

Supplementary Video 6

Supplementary Video 7

Supplementary Video 8

Supplementary Video 9

Supplementary Video 10
