# Supplementary material for: Behaviors of Glioblastoma Cells in in Vitro Microenvironments
Source: Sci Rep. 2019 Jan 14;9:85. doi: 10.1038/s41598-018-36347-7 (PMC6331579; doi:10.1038/s41598-018-36347-7)
Supplement: Supplementary file 1 — Supplementary Information [file 41598_2018_36347_MOESM1_ESM.pdf]

Behaviors of Glioblastoma Cells in *in Vitro* Microenvironments

Wenwen Diao[ab], Xuezhi Tong[c], Cheng Yang[d], Fengrong Zhang[d], Chun Bao[ef], Hao Chen[ef], Liyu Liu[g], Ming Li[bd], Fangfu Ye[bd], Qihui Fan[d]*, Jiangfei Wang[c]*, and Zhong-Can Ou-Yang[ab]*

[a]Key Laboratory of Theoretical Physics, Institute of Theoretical Physics, Chinese Academy of Sciences, 55 East Zhongguancun Road, Beijing 100190, China. E-mail: oy@itp.ac.cn
[b]School of Physical Sciences, University of Chinese Academy of Sciences, No.19A Yuquan Road, Beijing 100049, China
[c]Department of Neurosurgery, Beijing Tiantan Hospital, Capital Medical University, Beijing 100050, China Email: wjf1998@139.com
[d]Beijing National Laboratory for Condensed Matter Physics and CAS Key Laboratory of Soft Matter Physics, Institute of Physics, Chinese Academy of Sciences, Beijing 100190, China. E-mail: fanqh@iphy.ac.cn
[e]Wenzhou Institute of Biomaterials and Engineering, Chinese Academy of Sciences, Wenzhou 325001, China
[f]School of Optometry and Ophthalmology and Eye Hospital, Wenzhou Medical University, Wenzhou 325027, China
[g]College of Physics, Chongqing University, Chongqing 401331, China

Supplementary Material

Supplementary Images

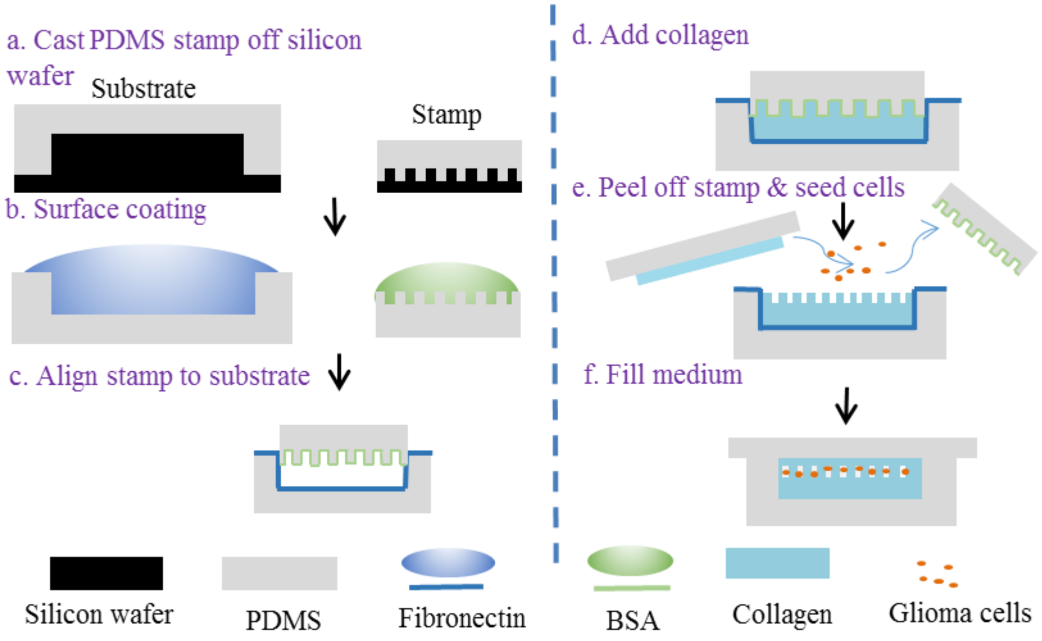

Supplementary Figure 1. Schematic of the method of the 3-D cell culture system. (a) PDMS

substrate and stamp were casted from the silicon wafer mould; (b) PDMS substrate surface was coated with fibronectin, and PDMS stamp surface was coated with BSA; (c) PDMS stamp was aligned with the PDMS substrate; (d) collagen was injected into the middle of the cavity; (e) PDMS stamp was unmoulded from the collagen, and cells were seeded into the gelled collagen micro-chambers; (f) collagen micro-chambers were covered with a gelled collagen top layer.

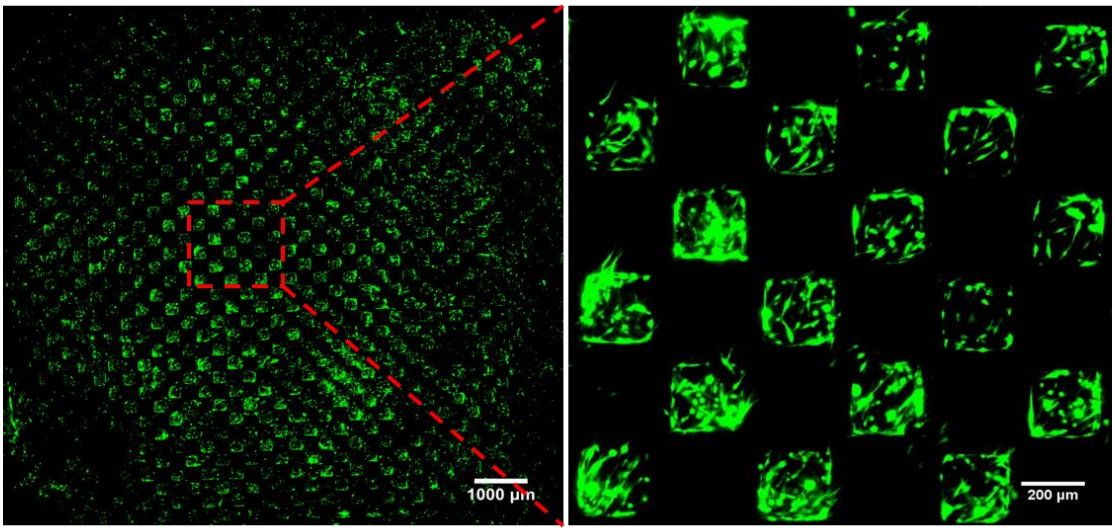

Supplementary Figure 2. (A) Overview of the micro-chambers on the whole collagen platform. (B) Enlarged image showing part of the micro-chambers array.

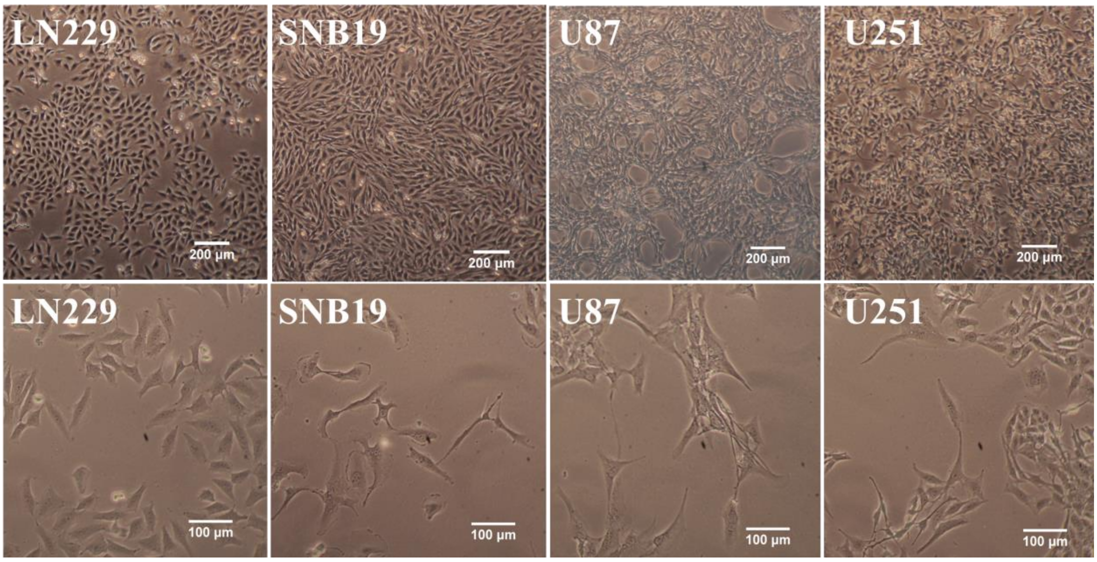

Supplementary Figure 3. The morphology of LN229, SNB19, U87, and U251 on Petri dish. In the traditional 2-D cell culture on a solid surface of Petri dish, LN229 cells show a regular triangular or diamond shape, with a flattened morphology; SNB19 cells have a long and thin shape, with the local cell orientations parallel to each other; U87 cells arrange as a network, and cells have a flattened and elongated morphology, possessing short filopodia[1]; U251 cells have an irregular shape, possessing long filopodia.

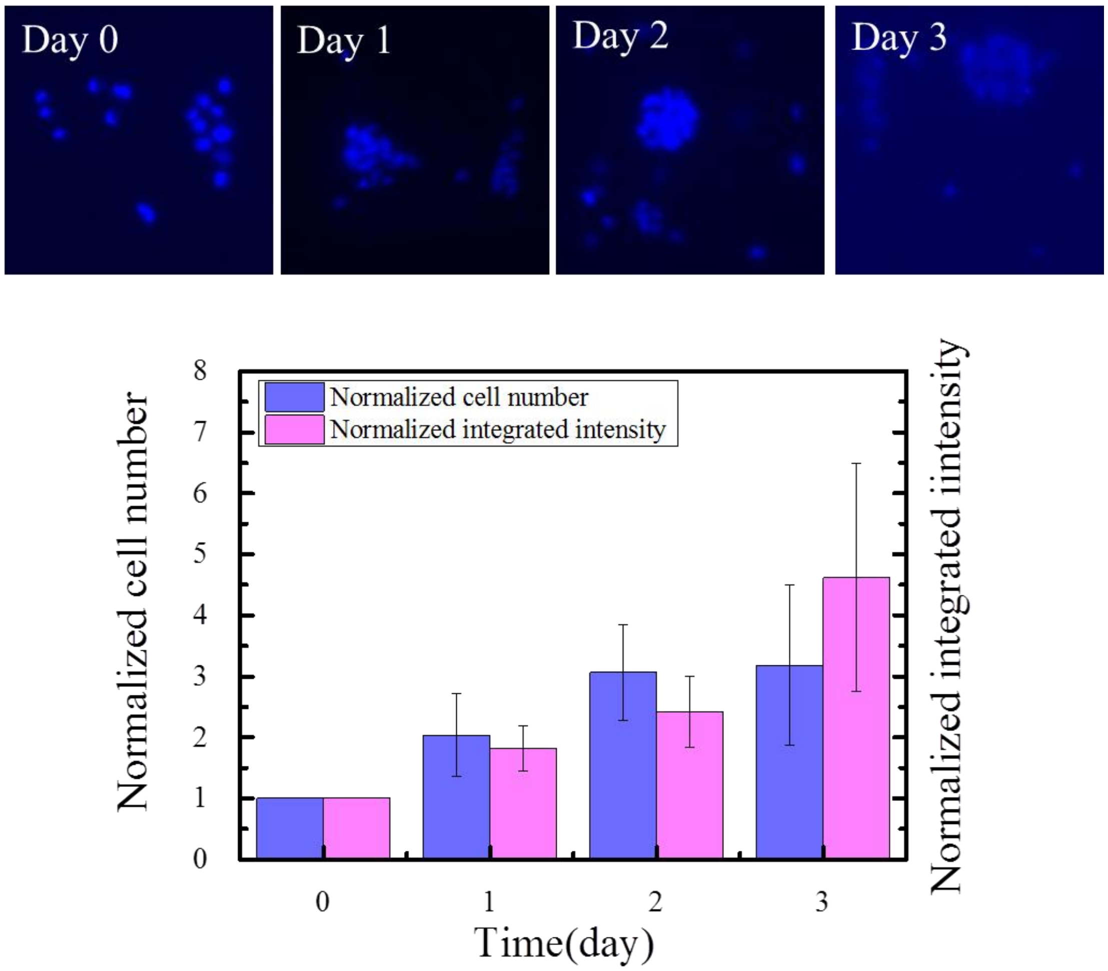

Supplementary Figure 4. (top) Images of nuclei staining and (bottom) relative increase of cell number, where the blue columns are values obtained by counting nuclei and the pink columns are values of the integrated intensities and both values have been normalized by their Day-0 results.

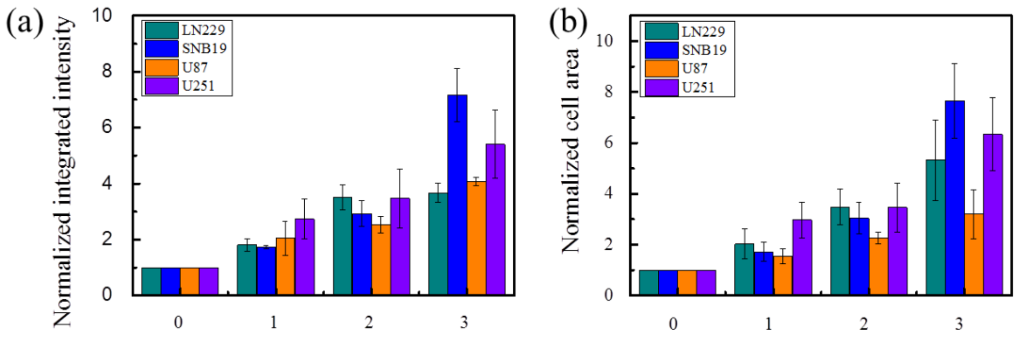

Supplementary Figure 5. Cell proliferation rate of LN229 (green), SNB19 (blue), U87 (yellow), and U251 (purple) indicated by (a) normalized integrated intensity; and (b) normalized cell area on Petri dish. Data presented as mean ±S.D. of three independent experiments.

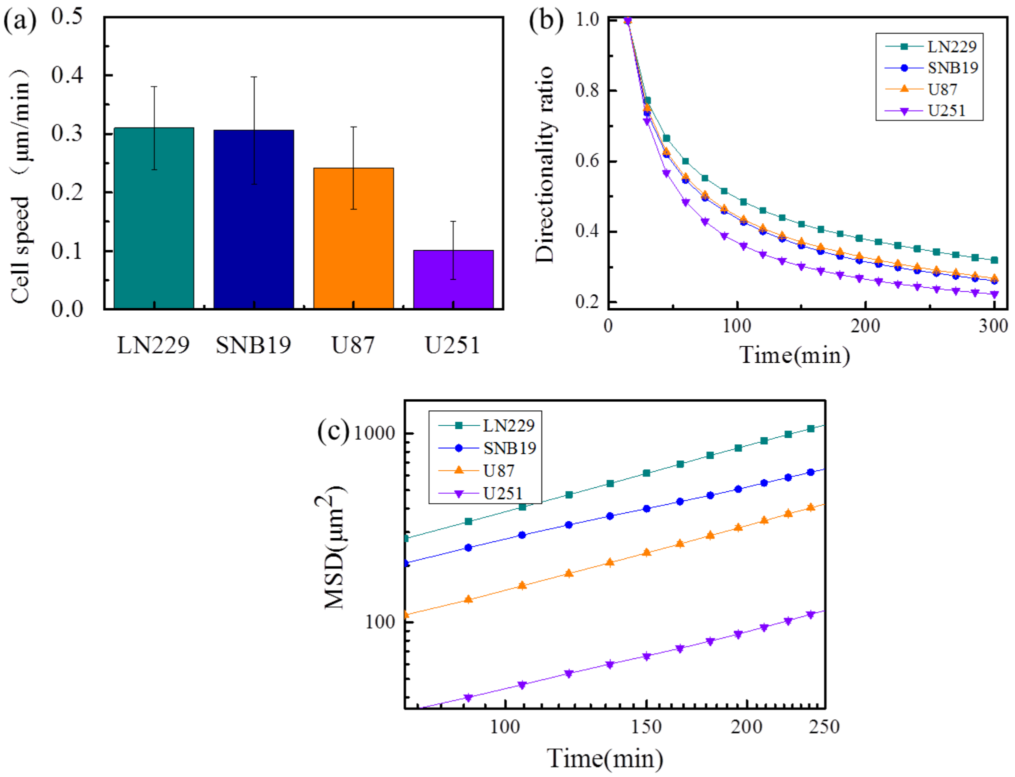

Supplementary Figure 6. Cell migration speed, directionality ratio, and MSD of four types of GBM cells on Petri dish: (a) the average cell migration speed (N = 200 for each test); (b) directionality ratio (N = 200 for each test); (c) mean square displacement (MSD), plotted as function of time interval (N = 200 for each test). Data presented as mean ±S.D. of three independent experiments.

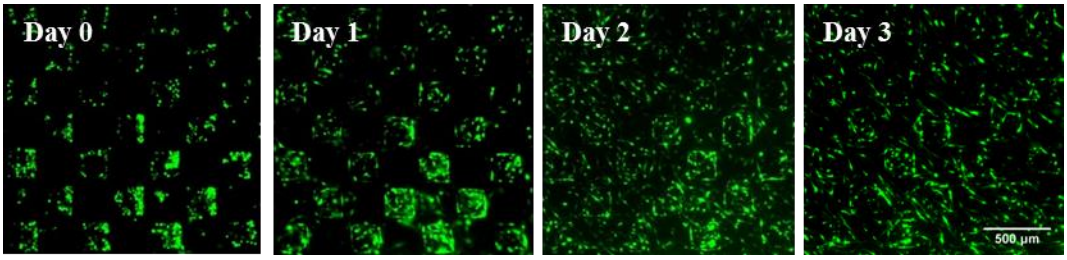

Supplementary Figure 7. SNB19 cell morphology in collagen micro-chambers for four consecutive days.

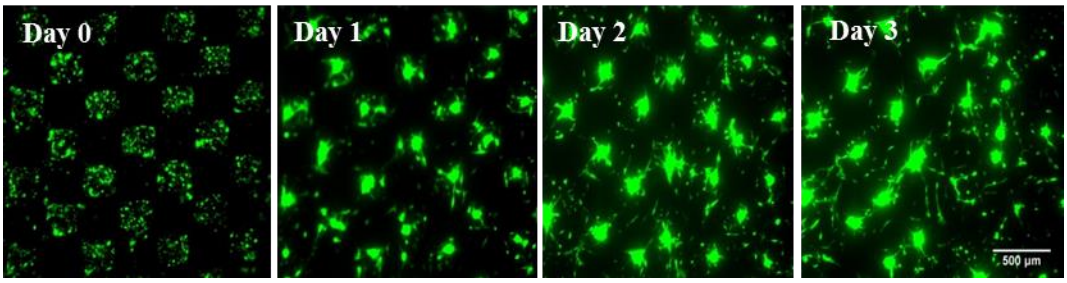

Supplementary Figure 8. U87 cell morphology in collagen micro-chambers for four consecutive days.

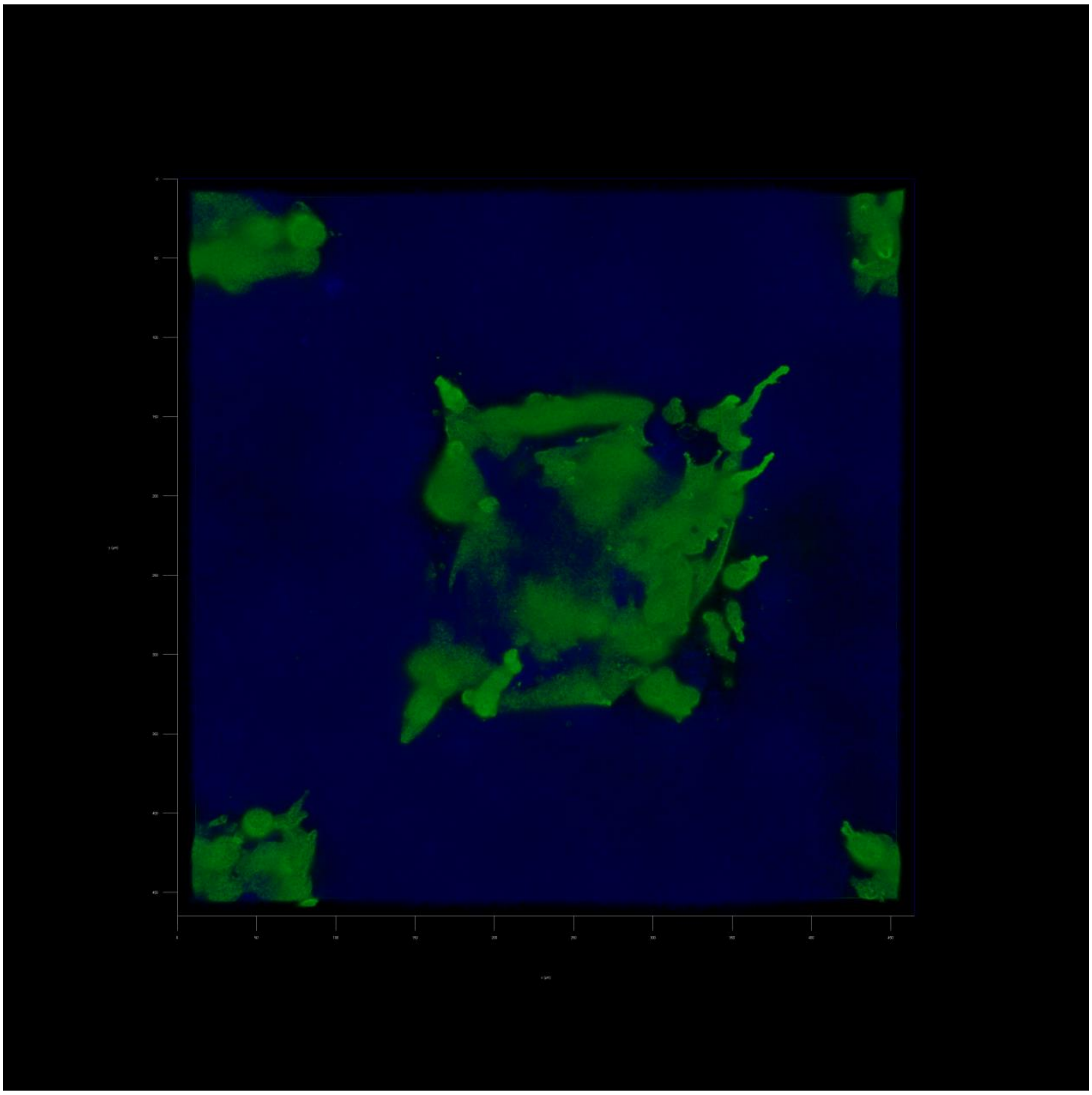

Supplementary Figure 9. Confocal images of LN229 cells in one micro-chamber on Day 5, where protrusions can be observed. The image shows 3-D structure in x-y plane sectioned from middle of the chamber along z-axis.

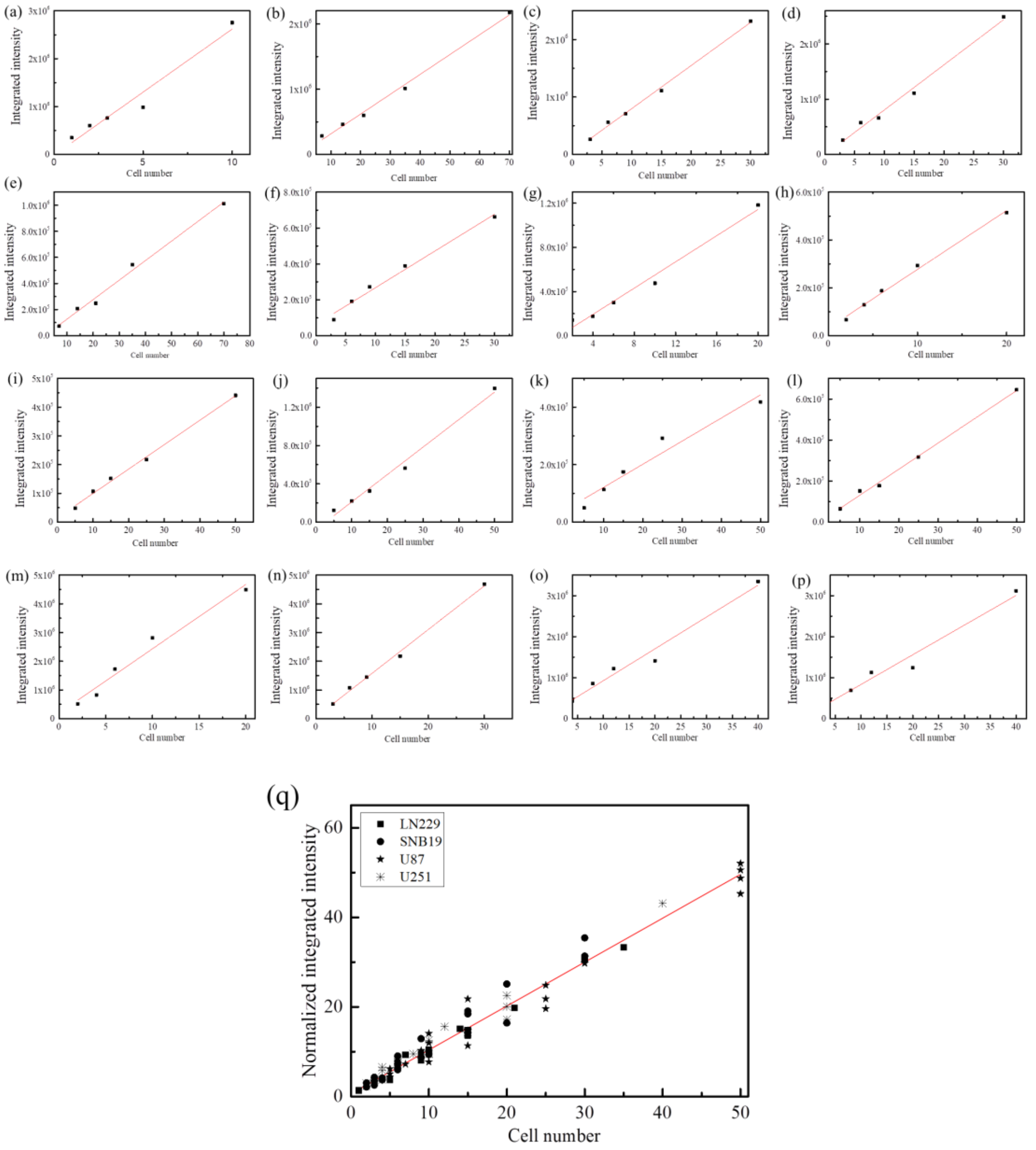

Supplementary Figure 10. Integrated intensities v.s. cell numbers on Day 0, for LN229 (a-d), SNB19 (e-h), U87 (i-l), and U251 (m-p). In (q), we plot for all four types of cells the relation between the cell numbers and the normalized integrated intensities, which were obtained by dividing the integrated intensities with the corresponding individual slopes of the fitting lines in (a)-(p), respectively.

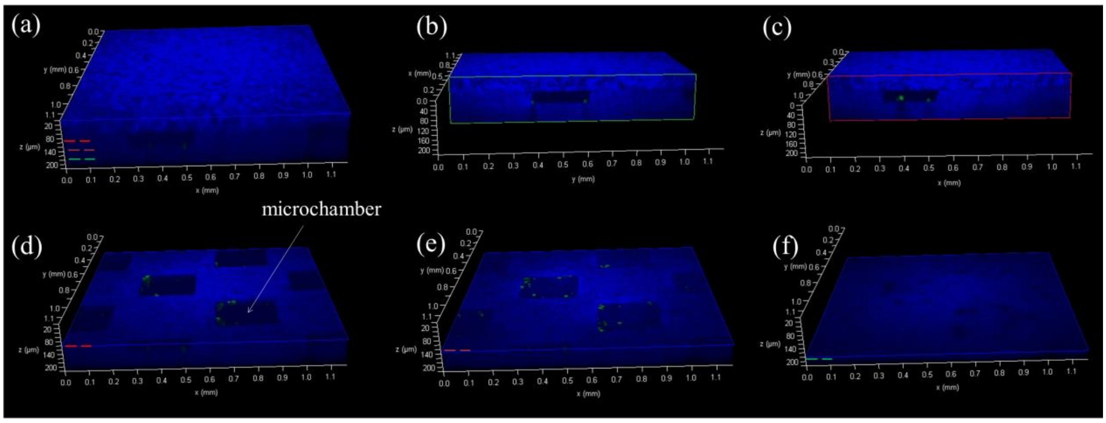

Supplementary Figure 11. Confocal images showing perfect micro-chambers on Day 0. (a) top-view; (b-c) side-view images; (d-f) top-view of sections at different z depths [corresponding to the red, pink, and green dashed lines in (a), respectively]. The collagen fibers (blue) were observed in the reflection mode. No crack exists between the collagen bottom and lid, and the micro-chambers were well sealed.

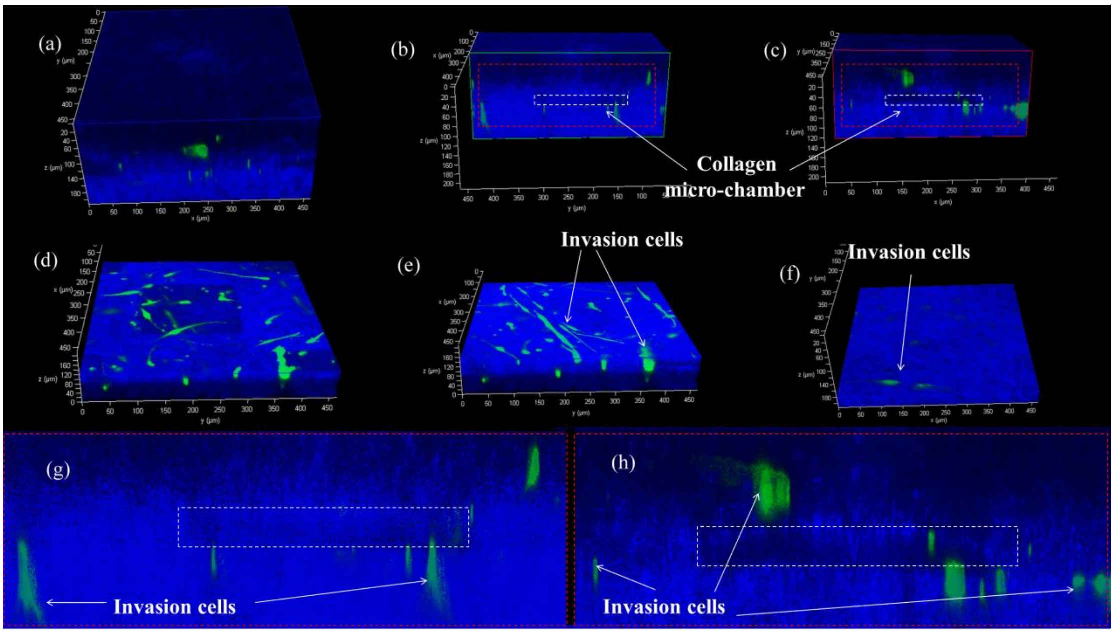

Supplementary Figure 12. Confocal images showing cell invasion of SNB19 cells on Day 3. (a) top-view; (b-c) side-view images, where the micro-chamber positions have been marked by dashed rectangles; (d-f) top-view of sections at different z depths. (g-h): Locally amplified images of (b) and (c). The collagen fibers (blue) were observed in the reflection mode. No crack exists between the collagen bottom and lid, and the micro-chambers were well sealed. Cells invaded into the gel along all directions, including into the collagen top and bottom. The chamber edges are not as clear as those on Day 0, due to the invasion of the cancer cells.

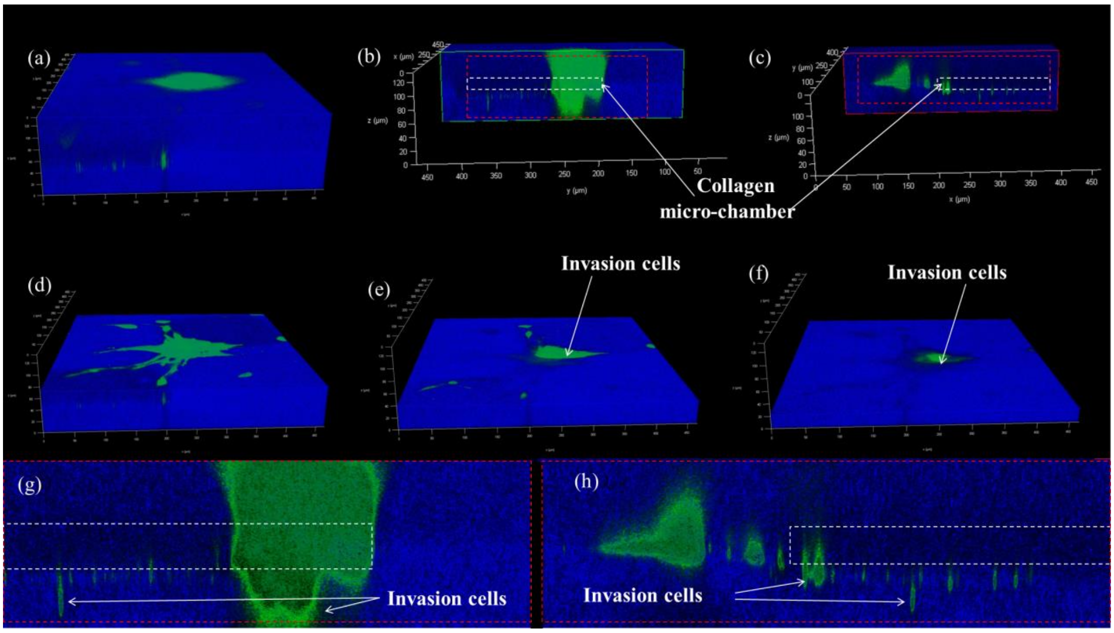

Supplementary Figure 13. Confocal images showing cell invasion of U87 cells on Day 3. (a) top-view; (b-c) side-view images, where the micro-chamber positions have been marked by dashed rectangles; (d-f) top-view of sections at different z depths. (g-h): Locally amplified images of (b) and (c). The collagen fibers (blue) were observed in the reflection mode. No crack exists between the collagen bottom and the lid, showing that the micro-chambers were well sealed. Cells invaded into the gel along all directions, including into the collagen top and bottom. The chamber edges are not as clear as those on Day 0, due to the invasion of the cancer cells.

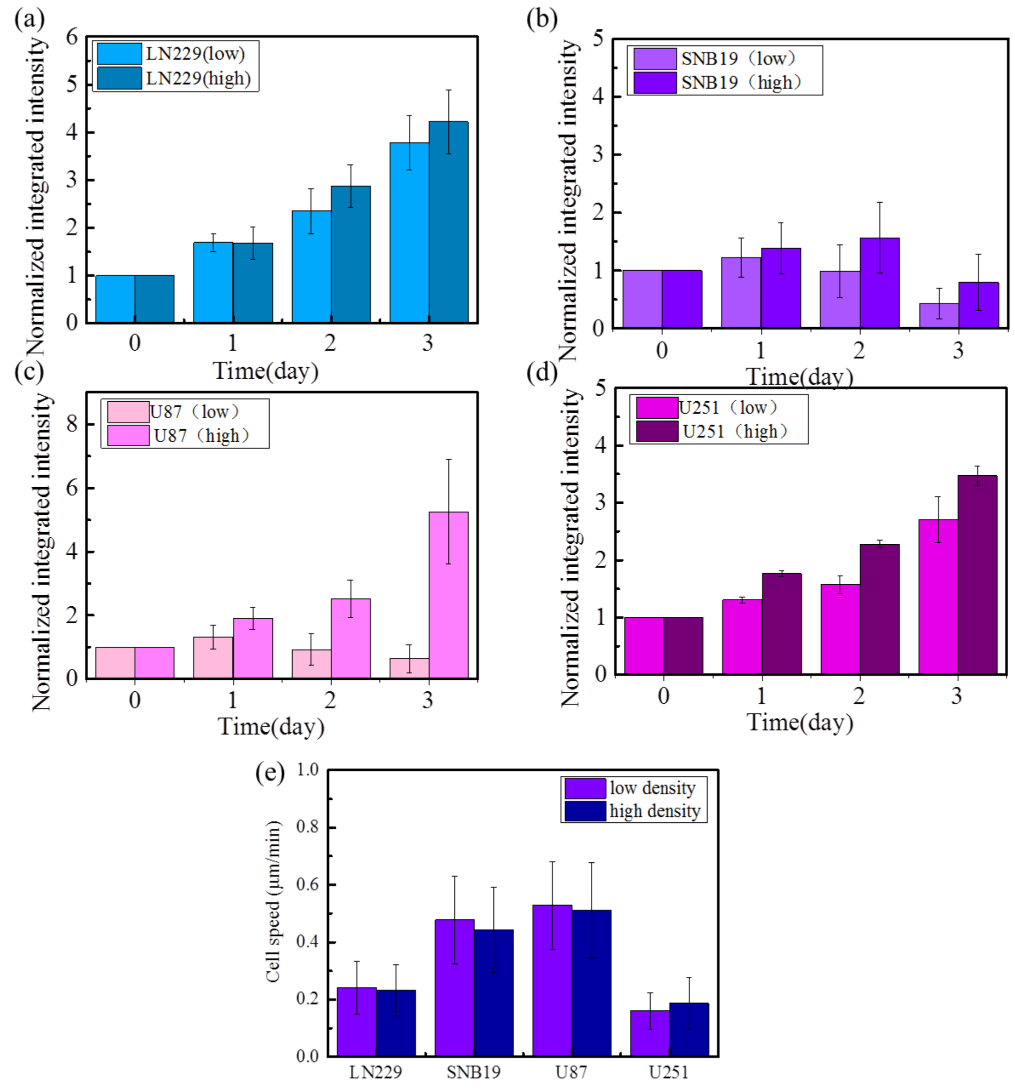

Supplementary Figure 14. (a-d) Cell proliferation rates for different cell densities in micro-chambers (with both the cells in individual chambers and those invading into the gel being counted); and (e) cell migration speeds (measured on the surface of collagen gel). The high density is about ~625 cells/mm^2, and the low density is about ~125 cells/mm^2.

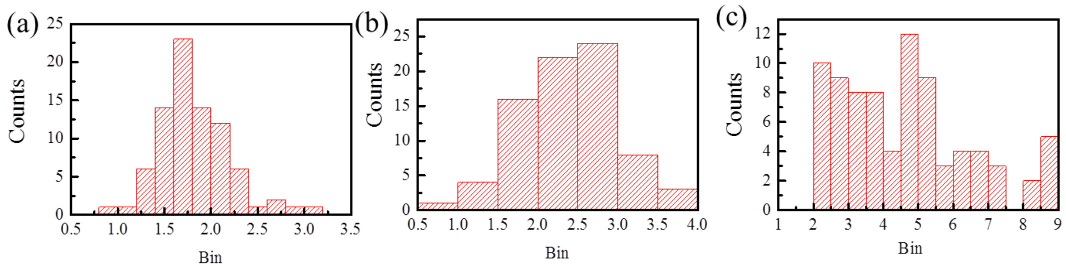

Supplementary Figure 15. Distributions of the cell number of U87 on different days. The distributions on Day 1 (a) and Day 2 (b) obey more or less a Gaussian-like distribution, but the distribution on Day 3 (c) does not.

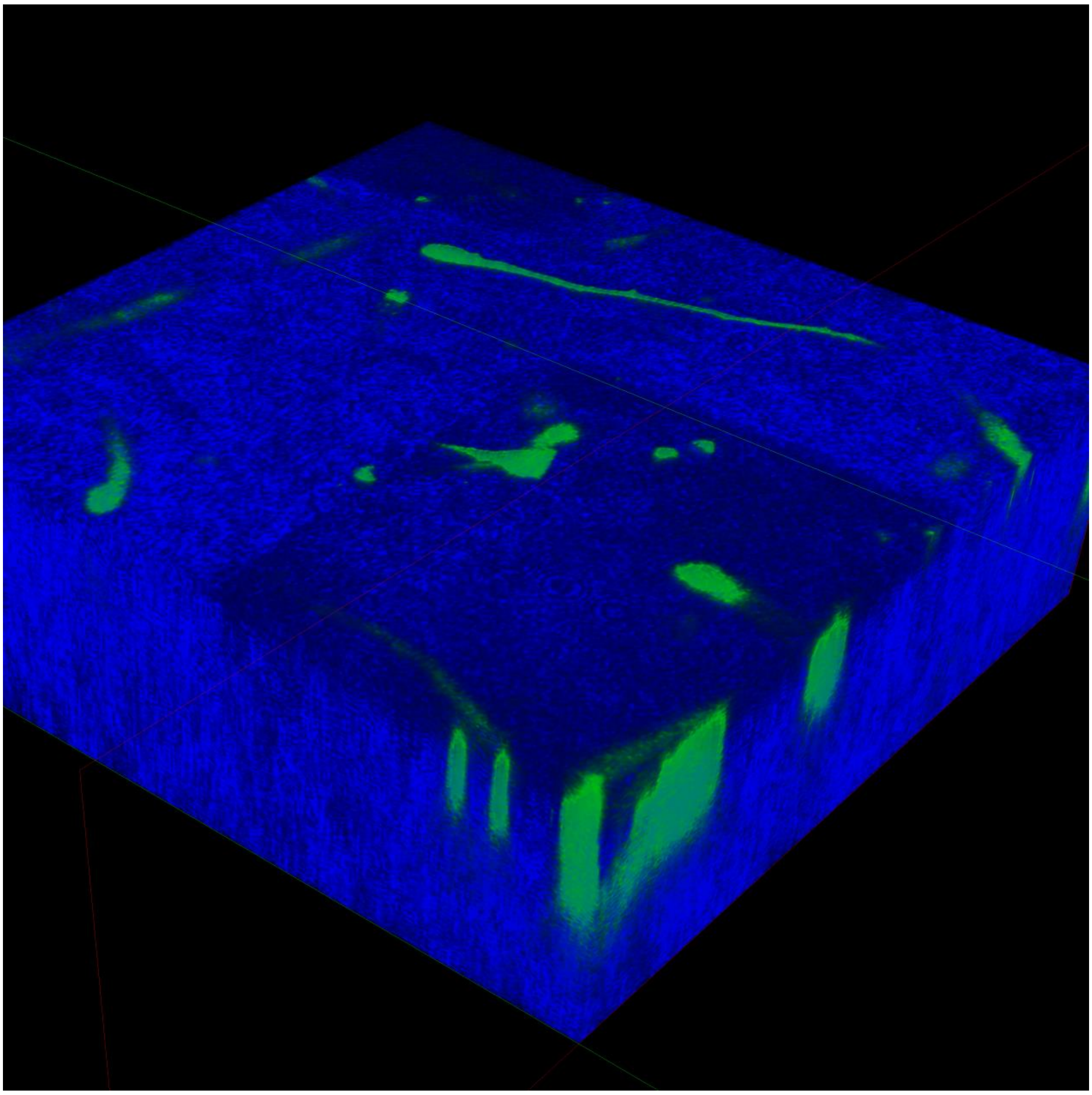

Supplementary Figure 16. Enlarged view of SNB19 cells invading into a micro-chamber, clearly showing that the SNB19 cells have invaded into collagen fibers.

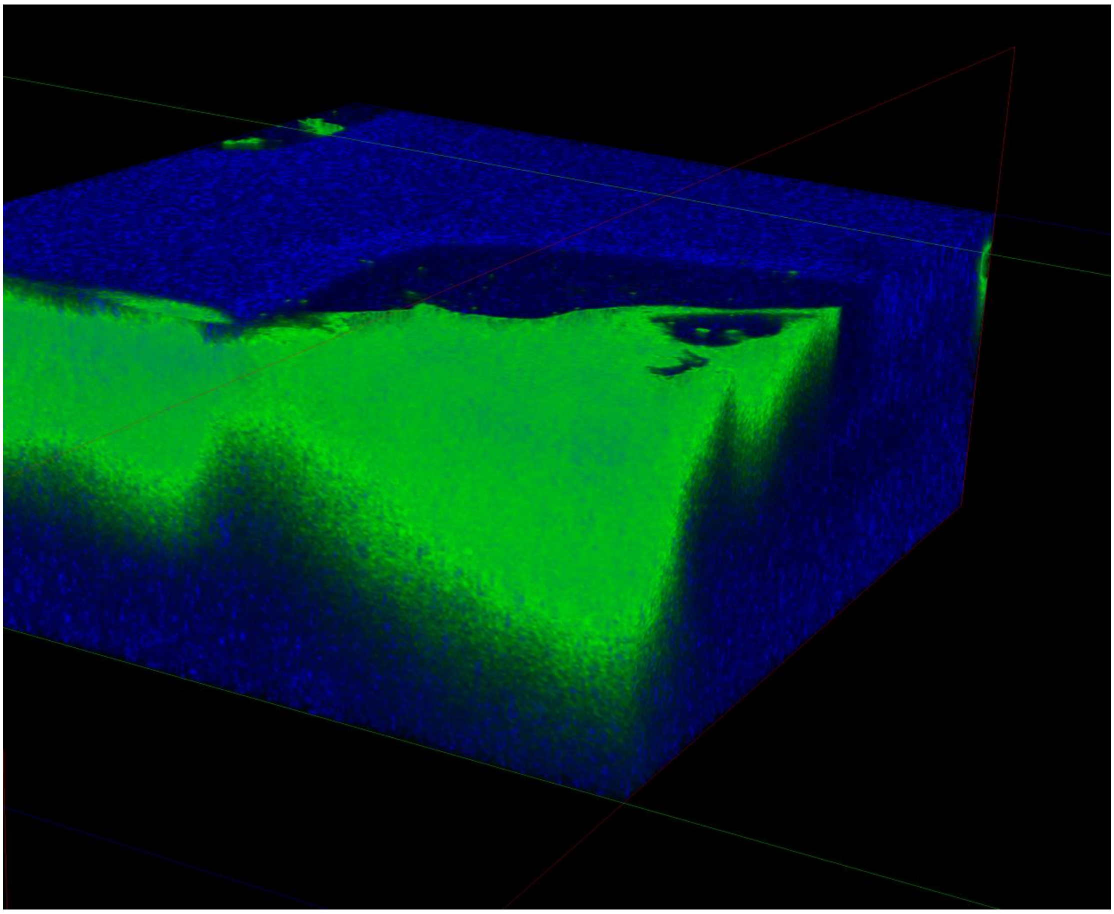

Supplementary Figure 17. Enlarged view of U87 cells invading into a micro-chamber, clearly showing that the U87 cells have invaded into collagen fibers.

Supplementary Videos

Supplementary Video 1. LN229 migration in one collagen micro-chamber on Day 1.

Supplementary Video 2. SNB19 migration in one collagen micro-chamber on Day 1.

Supplementary Video 3. U87 migration in one collagen micro-chamber on Day 1.

Supplementary Video 4. U251 migration in one collagen micro-chamber on Day 1.

Supplementary Video 5. Confocal images of SNB19 morphology in one collagen micro-chamber on Day 3. Many single cells invade into the surrounding collagen, even deep into the bottom of the collagen micro-chamber.

Supplementary Video 6. Images of U87 morphology in one collagen micro-chamber from confocal microscopy on Day 3. U87 cells form clusters and invade out of the micro-chamber in a collective pattern.

Supplementary Video 7. LN229 migration on a petri dish on Day 1.

Supplementary Video 8. SNB19 migration on a petri dish on Day 1.

Supplementary Video 9. U87 migration on a petri dish on Day 1.

Supplementary Video 10. U251 migration on a petri dish on Day 1.

1 Jiglaire, C. J. *et al.* Ex vivo cultures of glioblastoma in three-dimensional hydrogel maintain the original tumor growth behavior and are suitable for preclinical drug and radiation sensitivity screening. *Exp Cell Res* **321**, 99-108, doi:10.1016/j.yexcr.2013.12.010 (2014).
